# Where is the father? Challenges and solutions to the inclusion of fathers in child feeding and nutrition research

**DOI:** 10.1186/s12889-023-15804-7

**Published:** 2023-06-20

**Authors:** Andreia F. Moura, Kaat Philippe

**Affiliations:** 1 Department of Food, Nutrition and Dietetics, School of Biosciences, University of Nottingham, Sutton Bonington, UK; 2 Department of Management, Business and Social Sciences Faculty, MAPP Centre for Research On Value Creating in the Food Sector, Aarhus University, Aarhus, Denmark; 3 Centre des Sciences du Goût et de L’Alimentation, CNRS, INRAE, Institut Agro, Université de Bourgogne, Dijon, F-21000 France; 4 School of Public Health, Physiotherapy and Sports Science, University College Dublin, Dublin 4, Belfield, Ireland

**Keywords:** Fathers, Child feeding, Eating behaviour, Involvement, Recruitment methods, Gender ideology, Sociology, Psychology

## Abstract

Despite an increasing acknowledgement of fathers’ involvement in and impact on children’s lives, fathers remain underrepresented in child feeding and childhood obesity prevention research, interventions, and actions. Built on our own experiences with conducting research with fathers and recent evidence on this topic, this Research in Practice article has three aims. It will first substantiate the importance of including and studying fathers in the field of child feeding and childhood obesity prevention based on recent study results. Secondly, the article will present and discuss barriers to fathers' inclusion and participation (why isn't it happening?), among other issues, by drawing on sociological and gender ideological insights. Finally, it will provide recommendations and suggestions related to recruitment, focus and methods that can facilitate fathers’ involvement in future research, interventions, and practice. Taken together, this article aims to provide tools for giving fathers a voice in the field of child nutrition and by doing so, to decrease maternal-only “burden” of care. We hope our experiences and theoretical reflections will inspire and support researchers and practitioners to be as successful as possible in the realm of family care.

## Background

The increase in maternal employment in recent decades has resulted in a rise in fathers' caretaking responsibility [1]. Although mothers often remain the primary caregiver, fathers are involved in several aspects of child feeding, from decision making about the foods available in the home to the interaction with the child during mealtimes [2]. Yet, research studies targeting childhood obesity and feeding practices have primarily focused on mothers [3].

Systematic reviews on behavioural and public health actions revealed that fathers are substantially under-represented in childhood nutrition research and interventions [4, 5]. A systematic review of randomized control trials (RCTs) assessing interventions targeting childhood obesity identified that in studies requiring the participation of only one parent, fathers represented no more than 6% of all participants. When both parents were given the option to participate, 92% of RCTs did not report findings from fathers [5]. Similarly, an analysis of more than 600 studies on parenting and childhood obesity, revealed that 1% of those studies included fathers only, whereas 36% of studies included only mothers [4].

Among the several reasons explaining the underrepresentation of fathers in child feeding studies, there is the difficulty researchers face while recruiting men to take part in such studies, as scholars in the US [6] and Australia [3] reported. Indeed, Jansen et al. (2018) identified that some fathers can be reluctant in taking part in family-focused nutrition interventions even when they describe a high level of engagement with child feeding [3].

Faced with similar constraints when conducting studies with fathers for the EU project ‘Edulia: Bringing down barriers to children’s healthy eating’ [7], we developed this Research in Practice article.

Philippe, K investigated fathers’ and mothers’ involvement in child feeding related tasks at home (food planning, food shopping, cooking, feeding, or eating with the young child), their perceptions about their child’s eating behaviours, their feeding practices, and possible differences between mothers and fathers using surveys in France and Denmark. She also studied the links between child eating behaviours and mothers’ and fathers’ feeding practices in France and possible predictors of parental feeding practices in Denmark [8, 9]. In two other studies, she gathered quantitative and qualitative data of mothers and fathers related to parental portioning practices for pre-schoolers [10] and about their eating-related experiences during the COVID-19 pandemic in France [11, 12]. She experienced difficulties in finding fathers who were willing to participate in telephone interviews and it were mostly mothers who completed surveys received through the child’s school or day care centre. Via online panels, it was easier to reach fathers.

Moura, AF focused on barriers to healthy eating among new parents. While conducting qualitative studies the author had difficulties to find, contact and motivate fathers to participate in face-to-face interviews about (theirs and their families’) eating behaviours. In her first studies, investigating eating behaviours in the transition to parenthood in Denmark, France and Uruguay, the few men who accepted to be part of a 1-h interview were the ones whose female partners had persuaded them (those were female researcher colleagues) and fathers who also worked in academia or in the health sector [13, 14]. In another study, evaluating content posted on social media about feeding practices (focusing on sugar and sugary products), the author was faced with a complete absence of fathers in the online debates and forums about sugar consumption and health among children [15].

Built on our own experiences and recent literature on the topic, we have three goals in mind with this article. First, i.) we want to highlight the importance of fathers' inclusion in the field of child feeding and families’ healthy eating behaviours based on recent findings, then ii.) we want to describe and discuss barriers to fathers' inclusion and participation (why isn't it happening?), and finally, iii.) to offer recommendations and suggestions for future research/interventions/practice with fathers.

Considering published perspectives with relatable goals [16], this paper is an attempt to advance the discussion of the emerging work in the area by i.) drawing upon sociological and gender ideological paradigms, ii.) considering pre-conceptions of both researchers and fathers that explain the reluctance in participating/recruiting fathers to take part in research, and iii.) integrating new perspectives from European studies and other recent publications. A better understanding of the underlying factors acting as barriers to fathers’ participation in childhood obesity prevention actions and studies is crucial to advance scientific debates in this under-explored area and to provide hands-on solutions for researchers and practitioners.

## Findings and discussion

### Why is it important to include and engage fathers in child nutrition and health actions?

Fathers are getting more and more involved in several aspects of child feeding, from decision making about the foods available in the home to the interaction with the child during mealtimes [2]. Recent studies in France and Denmark confirm this trend, with fathers often being involved in grocery shopping and eating with the child [8, 9]. Although cooking is in most households still a task assigned to mothers [8, 17, 18], excluding fathers from child feeding research consequently ignores a possibly important actor and role model in a child’s life [16].

Most research on child feeding has been conducted with mothers and results obtained have often been used as a proxy for both parents [19]. This is problematic as differences seem to exist between mothers and fathers. Even though some studies found no significant differences in the frequency with which mothers and fathers use certain feeding practices for their child [20, 21], most studies find that fathers use coercive control practices (e.g., pressuring to eat, using food rewards) more often than mothers [8, 9, 22–25]. More research is warranted to investigate if these results from self-reported surveys also reflect what happens in practice at home. Nevertheless, these results may be cause for concern because these practices have been associated with counter-productive, negative effects on the child’s eating behaviour, both in mothers and fathers [8, 26].

The key role fathers play in their children’s eating behaviours goes beyond feeding practices. Fathers’ BMI, dietary intake, parenting skills, and food behaviours also influence their child’s weight status and eating patterns, including child’s intake of fruits and discretionary foods [19, 27–29]. Some of these findings were even observed after accounting for the effects of mothers [30]. Results of the “Healthy Dads, Healthy Kids” community Randomized Controlled Trial (RCT) suggest that as fathers change their eating patterns to include more fruits and vegetables there is a notable change in child eating patterns as well [31]. This evidence supports the hypothesis that the underrepresentation of fathers compromises the effectiveness of family interventions tackling childhood obesity [27].

### Barriers to fathers’ inclusion and participation

In the following, we discuss motives explaining why fathers might not take part in research on child feeding and nutrition practices (and some not even in feeding practices per se) and what researchers can do about it—including important reasons why academics should do so.

#### A reluctance to include fathers

One reason why fathers are not represented in child feeding research, is simply because they are not invited or actively recruited to participate in the studies. When over 300 fathers in the United States were asked why they believed men participate less than women in child health research, 80% mentioned it was because they were simply not asked [32]. A common argument for non-inclusion is that mothers take primary responsibility for feeding their child [33] or are considered the primary caregiver [34, 35]. This may indicate an underestimation of the role of fathers in children's development and children’s/family’s eating behaviour.

Knowledge about the difficulty to recruit and engage fathers may also act as a barrier to include them in feeding research, interventions, and actions. Why even invest efforts if there is little chance of success? Even though anecdotical and possibly not representative, when asked why fathers are not included in a new longitudinal study about child feeding in early childhood, a colleague in the field honestly stated that “it is easier to focus on mothers, as they are more motivated, and they will probably also be the one who will stay involved when parents would break up during the monitored period of the study”.

A reluctance of researchers to include fathers is however not the sole reason why fathers are not represented in child feeding and nutrition research. Some researchers make considerable efforts to include fathers in their studies, but face difficulties to find and convince fathers to participate [3, 6]. There is thus certainly also a reluctance from fathers’ side to participate, even when actively invited.

#### Fathers’ reluctance to participate, even when actively invited

When motivated to include fathers, a first challenge may be to find them, and secondly to “convince” those fathers to participate. We have encountered a few fathers who were reluctant to join the studies because they doubted their own capacities to make a valuable contribution to scientific studies on family/child’s eating. Those fathers were mostly blue-collar workers and expressed a certain discomfort to be “under the microscope” of researchers, whom they deemed to be “too knowledgeable” already to get any scientific insights from fathers with a lower socio-economic background. Another reason to question the value of one’s own contribution was the perceived lower involvement in child feeding compared to the child’s mother. Some fathers considered their female partners to be the “expert” in this regard, a pattern confirmed in other studies. For example, Mallan et al. (2014) identified that men who worked more hours perceived less responsibility for child feeding activities, and that fathers’ perceived responsibility for feeding their child was lower if the child was younger [36]. A study in Denmark also showed a lower perceived responsibility for child feeding in fathers compared to mothers, along with a lower feeding self-efficacy (e.g., a lower agreement with the statement “I can get my child to try veggies”) and lower cooking confidence (a lower agreement with the statement “I have knowledge and skills to prepare healthy meals for my family”) in fathers [9]. Fathers thus seem to question their own capabilities.

Furthermore, in interviews, fathers expressed a preference for interventions that can be delivered in a highly flexible and accessible mode, like online interventions [3]. This may reflect time-constraints as another barrier to fathers’ participation.

Both researchers’ reluctance to include fathers and fathers’ reluctance to participate and their lower confidence in their feeding capacities may be rooted in certain ideologies that are still present in our societies.

#### Identity and gender ideology as part of the problem

Men are commonly regarded as uninformed and unconcerned about dietary health unless they face major illness. This pattern is derived from a traditional masculine identity, in which risk-taking, invulnerability and endurance of pain are core values. Other factors accounting for men’s apparent obliviousness to nutrition issues are scepticism towards public health promotion initiatives and rejection of healthy foods that are portrayed as bland and slight [37]. As Gough & Conner (2006) suggest, these attitudes “can perhaps be linked to conventional masculinities which specify autonomous decision-making over obedience to authority, and plenitude and fulfilment over scarcity and self-denial” [37].

Conventional gendered traits manifest in parental practices within the family realm. Studies have shown that the role of nurturing remains related to a maternal identity [38, 39]. For fathers, childcare is expressed by assuming a playful role and by facilitating processes of autonomy in their children. Therefore, fathers are more willing to participate in outdoor activities where they can teach independence and can encourage risk taking—for example, by motivating the child to climb the playground a bit higher and to overcome downfalls quickly [40].

Reasons for gender differences in childcare include residues of gendered upbringing and cultural aspects that mark mothering and fathering as inherently distinct identities. Reminiscent gendered stereotypes from a past where fathers assumed the breadwinner role whereas the mothers performed the domestic caregiving tasks, still prevail [40]. Moreover, archaic discourses on women’s supposed biologically predisposition for childcare might contribute to maintain gendered practices among heterosexual couples [39].

Traditional gendered patterns also seem to be reinforced by what theorists have defined as “maternal gatekeeping” [41]. This concept implies that women might actively exclude men from domestic activities and resist to give up the role as the main caregiver; a role in which they can exercise power and expertise. At the same time, men expect women to take this role and, therefore, might not make efforts to acquire childcare skills. In fact, the lead that mothers take is deeply rooted in the moral duty to care felt by women, whereas men’s moral responsibilities lie in a commitment to financially provide for the family [40]. Women might also take up this central role on behalf of a child's health. When the male partners are less health conscious, they can undermine attempts to child (healthy) feeding [38]. For mothers, an emotional dimension is also involved: they perceive domestic food work as an emotional support in the crystallisation of family ties, while fathers perceive it as a simple task detached from any emotional dimension [42].

Anyhow, patriarchal ideologies contribute to men’s greater power to decide the levels and parameters of their involvement in family life, including the engagement in food and feeding practices [38, 39]. In this regard, men’s domestic cooking is described as usually a chosen form of leisure (pleasure-oriented), commonly practiced on the weekends, therefore distant from day-to-day care obligations [43]. In contrast, mothers can perceive cooking as a pleasurable activity but also a difficult task or burden, depending on the context, e.g., with whom, when [44, 45]. Mothers can moreover struggle with combining the wish to accommodate family members’ food preferences when cooking and normative injunctions: “between self and others”, but also “between health and pleasure” [46]. These ideas are supported by recent findings from a qualitative COVID-19 study [12]: French fathers mostly appreciated the available time during the lockdown for additional cooking, while mothers perceived it both as a pleasure and a burden to prepare these additional meals. Mothers also struggled with the balance between pleasure and “too much” pleasure in terms of preparing and consuming “tasty” food in the family during the lockdown.

Scholars have denounced the moral burden posed on mothers as solely responsible for children’s body weight and wellbeing in public health initiatives [47–49]. In the debates and policies targeting childhood obesity, there is inadequate recognition of the unequal division of responsibility for child feeding and care [49, 50]. Childhood obesity is commonly portrayed as an outcome of women’s failure, whereas men are not taken accountable or responsible for their child’s health [48]. Therefore, it is timely to include men in food and nutrition interventions and research, even when they are *not* engaged in child feeding. By inviting men to take part, researchers recognize and shed light on fathers’ crucial influence and responsibility for their child’s health and care.

By excluding fathers from child feeding and nutrition studies, researchers contribute to neglect and silence men’s responsibility in promoting child health and in family food work. This is reflected in the number of studies addressing ‘parental’ influence on children's eating patterns that focus solely on mothers, thus positioning mothers as the ‘default parent’. As a result, there is a reinforcement of gendered ideologies and a notable lack of knowledge on fathers' influence over children’s diet, weight, and health [51, 52].

Researchers have thus an important role in disrupting the pervasive silence around fathers in childhood obesity discourses. We believe that including men in child feeding studies can be an important step to create necessary knowledge, to decrease the burden of maternal responsibility, and to motivate the adoption of domestically responsible masculinities.

### Strategies for promoting fathers’ participation in child feeding research and actions

**Fig. 1:**
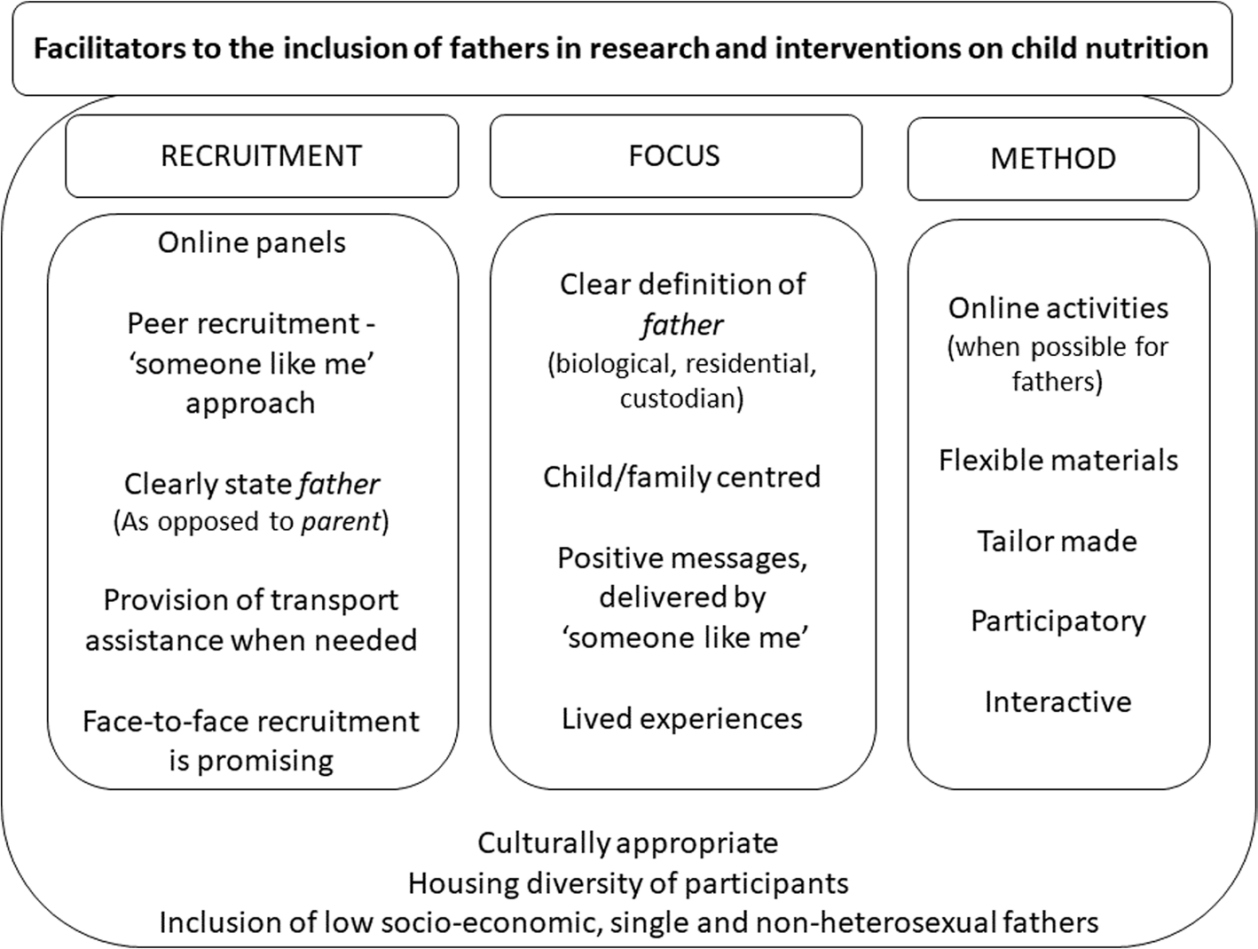
Facilitators for the inclusion of fathers in research and interventions targeting child’s nutrition and health: strategies for recruitment, focus and method, based on evidence of the literature

In this last part, we describe own experiences with working with fathers in Europe, combined with those of other researchers who have worked with fathers in the last years (mostly in North America and Australia). Based on these experiences, we summarize recommended practices in Fig. 1.

We tried different strategies to recruit, include, and persuade fathers (via schools and day-care centres, online panels, snowball sampling) to participate in (paper and online) surveys, qualitative studies (face-to-face and telephone) and an intervention. When aiming at including a high number of fathers (e.g., data collection via surveys), it was, from our experience, most time efficient and effective to recruit them via national online panels. To illustrate, 141 French fathers [11] and 321 Danish fathers [9] completed an online survey on child feeding in a time span of one to two weeks when recruited via online panels, compared to reaching only 169 French fathers in a different study after months of intensive recruitment via local schools, day-care centres and national online groups [8]. The fact that no incentive was involved in the latter study may possibly also have played a role. This aside, many men are registered in various countries for completing online surveys in exchange for points or gift vouchers and actively contribute. Fathers recruited via online panels were also willing to contribute to qualitative research, in our experience. In an earlier study where Danish fathers were approached via snowball sampling, only 3 fathers participated [13], whilst recruiting through online panels resulted in the participation of 15 fathers in a later study [53]. Downsides of this approach via online panels though, may be participation bias [54] and inattentive response behaviour [55]. Implementing techniques to detect respondents’ inattention [56] and the application of data cleaning (e.g., excluding participants based on unrealistic response times [57]) are thus recommended in this case.

Our experience is partially aligned with what other researchers have reported. A study with 436 Australian fathers from a broad range of educational backgrounds showed that the vast majority of those fathers were willing to participate in research studies on child feeding. Most of the fathers also agreed that they should share equal responsibility with mothers in child feeding [36]. However, other studies reported that engaging fathers of very young children (i.e., infants) in child feeding interventions may be more difficult than involving fathers of older children [52, 58].

Another study with Australian fathers indicated a preference for family-focused and technology-based (online) interventions, focusing on the family and how to make changes at the family level, as opposed to individual, group or fathers-only approaches [3]. This corroborates what we observed in a family-based intervention, where 15 fathers (recruited from an online panel) were willing to participate [53]. In ours and in the Australian study, online delivery of information (using Apps, video chats, etc.) was preferred due to convenience and possibility to share the information with the whole family. The same Australian fathers also reported low commitment to participate in nutrition interventions outside working hours. Yet, they were not very keen on participating in family-focused nutrition interventions in the workplace [3]. To increase fathers’ participation, the researchers of this study emphasized the importance of the use of comprehensive and high-intensity recruitment strategies (e.g., email, telephone call, face-to-face contact). They indicated that face-to-face recruitment was a particularly helpful strategy, which is corroborated by Mitchell et al. (2007), who asserted that face-to-face interaction provides an opportunity for participants to meet the researchers and to understand how much their participation is valued [59].

The authors of another study in Australia (with a large group of fathers (*N* = 436)) emphasize the importance of “first evaluating fathers’ preferences for interventions before developing and delivering culturally appropriate interventions” [3]. This seems to be particularly important to ‘hard to reach’ populations, which seems to be the case of blue-collar working fathers. Interventions that increase fathers’ self-efficacy in supporting the family, rather than targeting men’s own health, are promising, when considering fathers with low socio-economic backgrounds. In fact, fathers seem more willing to participate in programs that have their children as the focus [3, 60, 61]. An earlier study with limited-income, urban fathers also suggested that effective nutrition education with this target group should focus on food (as opposed to complex nutrition concepts), basic information, positive messages delivered in a positive way, while honouring the diversity of participants [60].

Cultural adaptation, using recruiters and researchers who are representative of the social and cultural background of the targeted group seems important. Urban African American fathers pointed out word-of-mouth and advertising in a fashion they could relate to as attractive recruiting strategies. Those fathers expressed that hearing about parenting programs from other fathers like themselves (‘someone like me’ effect), in addition to the provision of transportation assistance and incentives would motivate them to join parenting interventions [62].

To maximize success in reaching fathers, peer recruitment has been reported as an effective strategy [62], as well as forming collaborative relationships with community stakeholders or key contacts [32]. Recruiting fathers through the target child in educational institutions (e.g., kindergarten, elementary schools, nursery) has also been successful [19, 59]. In any case, recruitment venues should be chosen based on fathers’ interest and characteristics. For example, findings from a qualitative study with US fathers indicate that barbershops can be effective recruitment venues for non-white fathers, while doctors’ offices may be more effective for recruiting white fathers (for paediatric studies) [32]. US fathers also expressed that they were more likely to participate in paediatric research when the expected time commitment is small, when the benefits of participation are specified, and when the recruitment materials clearly state father’s involvement (as opposed to the more generic wording “parents”) [32]. From our experience, this was especially the case when it was clearly stated that we were interested in *both* parents’ point of view (all caregivers fulfilling a parent role were eligible – taking into account different family structures). Therefore, we purposefully handed out two questionnaires to parents.

The majority of studies targeting both parents usually recruit mothers, who are then asked to approach their partners [36, 59]. Although this strategy might be effective, it can restrain the diversity of fathers joining nutrition programs, excluding single and non-heterosexual fathers and men who are not in a positive relationship with their female partners [3]. Scholars agree that the involvement of fathers in great numbers and diversity, including fathers from varied ethnic groups, nationalities, socio-economic status, sexual orientation, family statuses and structures, is paramount for family-based obesity research [63]. Equally important is to take into account the operational definition of “father” that is relevant for the study, and to adequately describe the fathers’ biological, residential and custodial status in the occasion this information is of relevance for the analysis and research goals [32, 59].

Insights from the conference “Engaging the Forgotten Parent: Conference of Experts on Fathers’ Role in Children’s Weight-Related Behaviours and Outcomes” indicate the following strategies to be helpful for recruiting, engaging and maintaining fathers’ participation in family-based obesity research: i) the use of flexible materials tailored to fathers’ needs and interests, including lived experiences of fatherhood; ii) interventions delivered in an interactive format, preferably by a facilitator with whom fathers can identify (e.g., also a father with similar age, social background, marital and custodial statuses) [63]. A way to reach this, could be by completing participatory research with fathers, which means that we actively involve them in the research, beyond providing data and instructions [64]. Fathers – as “experts” alongside researchers – can be involved in the development of the entire research design, the design of methods, data collection, data analysis, and the dissemination of findings. This way, we make sure that fathers’ voices are heard, materials and methods adapted to their needs and interests, and to ensure culturally and/or socially appropriate and sensitive research that is both relevant and equitable [65]. This will likely have a positive impact on the research outcomes. Moreover, this participatory research with fathers may be of particular importance in the field of child and family nutrition, as researchers in this field, like ourselves, are most commonly female. Fathers’ and men’s input can shed light on elements in the design and conduct of studies and interventions that may otherwise possibly be overlooked or under/overestimated.

To summarize, we unite the facilitators for including fathers in research and interventions on child nutrition in the above figure (Fig. 1).

As a final note on more general recommendations, our experience with fathers indicated that preconceptions about the research work and the researchers themselves might turn the participation in scientific studies into an intimidating experience (especially among fathers with lower SES). In this regard, we suggest engaging in activities that bring science and scientists closer to the public, such as the European researchers’ night. This is a yearly, Europe-wide event open to people of all ages, which showcases diverse research projects at different locations in an understandable way, gives insight in researchers’ work life, and the impact of their research on citizens' daily lives. This type of event and other public research activities, such as open science days and public and patient involvement activities of universities and research centres, generally spark people’s interest in research, “humanize” researchers and their work, and can motivate people to actively participate in research initiatives. The more accessible and trusted research becomes, the easier it will likely be to motivate people to participate and become engaged.

## Conclusions

Engaging fathers in the prevention of childhood obesity is critical, due to their influence on their child’s eating behaviours as well as the role they play in family (healthy) eating. Yet, research and interventions on child feeding and families’ eating behaviours lack the representation of fathers, for reasons related to the fathers themselves (e.g., reluctance to participate due to low self-efficacy, gender ideology) and researchers (e.g., underestimation of the role of fathers in children's eating, methodological concerns). By reflecting upon our experiences (as health and social researchers) and drawing upon the evidence in the field, we propose strategies to increase fathers’ participation and engagement throughout different stages: recruitment, design and focus of material/approach and methods to be applied. We hope the suggestions presented here can inspire and encourage scientists and practitioners to include fathers in their approaches to child/family’s healthy eating. This is of great importance for several reasons: childhood obesity prevention and management, father’s health, participatory fatherhood, and to disrupt pervasive discourses of maternal (sole) responsibility for their families’ health.

## Data Availability

Not applicable.

## Abbreviations

BMI: Body Mass Index
SES: Socio-economic status
RCT: Randomized Controlled Trials
